# Examining Participant Engagement in an Information Technology-Based Physical Activity and Nutrition Intervention for Men: The Manup Randomized Controlled Trial

**DOI:** 10.2196/resprot.2776

**Published:** 2014-01-03

**Authors:** Camille E Short, Corneel Vandelanotte, Marcus W Dixon, Richard Rosenkranz, Cristina Caperchione, Cindy Hooker, Mohan Karunanithi, Gregory S Kolt, Anthony Maeder, Hang Ding, Pennie Taylor, Mitch J Duncan

**Affiliations:** ^1^Center for Physical Activity StudiesInstitute for Health and Social Science ResearchCentral Queensland UniversityRockhamptonAustralia; ^2^Kansas State UniversityDepartment of Human NutritionKansas State UniversityManhattan, KSUnited States; ^3^University of British ColumbiaFaculty of Health and Social DevelopmentUniversity of British ColumbiaKelowna, BCCanada; ^4^The Australian eHealth Research CenterICT CenterCSIROHerstonAustralia; ^5^University of Western SydneySchool of Science and HealthSydneyAustralia; ^6^University of Western SydneySchool of Computing, Engineering, and MathematicsSydneyAustralia; ^7^Food and Nutritional SciencesCSIROAdelaideAustralia

**Keywords:** qualitative, intervention, physical activity, retention, technology, website, engagement

## Abstract

**Background:**

Males experience a shorter life expectancy and higher rates of chronic diseases compared to their female counterparts. To improve health outcomes among males, interventions specifically developed for males that target their health behaviors are needed. Information technology (IT)-based interventions may be a promising intervention approach in this population group, however, little is known about how to maximize engagement and retention in Web-based programs.

**Objective:**

The current study sought to explore attributes hypothesized to influence user engagement among a subsample of participants from the ManUp study, a randomized controlled trial testing the efficacy of an interactive Web-based intervention for promoting physical activity and nutrition among middle-aged males.

**Methods:**

Semistructured interviews were conducted and audiotaped with 20 of the ManUp participants. Interview questions were based on a conceptual model of engagement and centered on why participants took part in the study, what they liked and did not like about the intervention they received, and how they think the intervention could be improved. Interview recordings were transcribed and coded into themes.

**Results:**

There were five themes that were identified in the study. These themes were: (1) users’ motives, (2) users’ desired outcomes, (3) users’ positive experiences, (4) users’ negative emotions, and (5) attributes desired by user.

**Conclusions:**

There is little research in the field that has explored user experiences in human-computer interactions and how such experiences may relate to engagement, especially among males. Although not conclusive, the current study provides some insight into what personal attributes of middle-aged males (such as their key motives and goals for participating) and attributes of the intervention materials (such as usability, control, and interactivity) may impact on user engagement in this group. These findings will be helpful for informing the design and implementation of future health behavior interventions for males.

**Trial Registration:**

Australian New Zealand Clinical Trials Registry: ACTRN12611000081910; https://www.anzctr.org.au/Trial/Registration/TrialReview.aspx?ACTRN=12611000081910 (Archived by WebCite at http://www.webcitation.org/6M4lBlvCA).

## Introduction

### Engaging Men in Healthy Lifestyles

Despite strong evidence that physical inactivity and poor nutrition are associated with an increased risk of chronic diseases and mortality [1,2], the majority of the adult population in western societies continues to live an unhealthy lifestyle [3-7]. Interventions that promote healthy lifestyles at a population level are needed to address this public health issue [1,8]. Of particular concern is how to engage males in such interventions. Compared to their female counterparts, males are less likely to engage in lifestyle modification programs, utilize health services, and participate in intervention research [9,10]. This may be a contributing factor to the shorter life expectancy and higher age-specific rates of chronic diseases, such as heart disease, Type 2 diabetes, obesity, and mental health issues experienced by males compared to females [11-13]. To effectively reach males and have them engage in health promotion initiatives, interventions that appeal to males and meet their specific needs are required.

There is a clear gap in the research literature addressing the physical activity and nutritional behaviors of males. Little male-specific research has been undertaken, and best practice approaches have not been identified [12,14,15]. Overall, males receive less educational advice than women from health professionals about modifiable lifestyle behaviors associated with disease risk [16], and there are a lack of resources available that are targeted specially at males [17,18]. There is strong evidence that behavior change interventions that address characteristics and behavior change determinants unique to particular subgroups are more effective than generic “one-size-fits all” approaches [19]. Given the difference in social norms regarding masculine and feminine gender roles and the influence of these norms on behavior [16], the development of resources targeted specially at men is required.

### Purpose of the ManUp Study

The purpose of the ManUp study [20] was to address this issue by developing and testing innovative physical activity and nutrition modification strategies that would be appealing and effective for middle-aged males. As part of this study, comprehensive reviews of the literature were undertaken to identify strategies that have worked in previous interventions targeting males [21,22]. Original research exploring male’s intervention preferences [23] and factors influencing physical activity and nutrition behavior among males were also conducted [24]. From this, Web- and mobile phone-based interventions that include the delivery of quantitative information and feedback and encourage goal setting and self-monitoring were identified as a promising intervention approach for targeting and engaging males. This was due to Web-based interventions with these features successfully improving physical activity and nutrition behaviors in previous studies [21,22], and the potential for Web-based interventions to be accessed by large numbers of individuals without time of day or geographic restrictions, which was considered necessary for overcoming key barriers to participating in healthy lifestyle behaviors reported by males (such as work and family commitments) [24]. On this basis, a Web-based social cognitive [25,26] and self-regulation theory-based [27] intervention, designed to increase knowledge and self-efficacy via information provision, goal setting, and self-monitoring, was developed and evaluated in a two-arm randomized controlled trial [20].

### Issues With Participant Engagement and Retention

Despite the comprehensive and systematic approach to intervention development, issues with engagement and retention of participants were experienced. Few participants logged on to the website regularly (median number of log-ins over the 9 month intervention period was 2 per participant) (interquartile range-IQR=6), and engagement in key behavior change components was low overall (median number of self-monitoring entries was 1 per participant) (IQR=20); median number of challenges initiated was 1 per participant (IQR=3). The dropout was high across both intervention groups, but was significantly higher in the Web-based intervention arm (52/96, 54%, had dropped out at the 9 month follow-up) compared to the print-based positive intervention control arm (96/205, 46.8%, had dropped out at the 9 month follow-up). This is in line with findings from other Web-based-delivered health behavior intervention studies [14,28]. If such interventions are to be effective public health tools, a greater research focus on user engagement is needed [28,29]. This should include research examining the underlying theoretical mechanisms of engagement.

Prior research examining user experiences in human-computer interactions suggests that engagement consists of four distinct stages: (1) the point of engagement, (2) a period of sustained engagement, (3) disengagement, and (4) (possibly) reengagement [30]. According to O’Brien and Toms’ [30] conceptual framework of engagment with technology, the *point of engagement* is initiated by the aesthetic appeal and/or novel presentation of the interface, the users' motivations and interests, the users' ability and desire to be situated in the interaction, and to perceive that there is sufficient time to use the application. At this phase, users typically have a goal in mind for what they would like to gain from the interaction. Engagement is sustained when users are able to maintain their attention and interest in the application and is characterized by positive emotions. Attention and interest are perpetuated by the interactivity of the computer environment (physical, social, and cognitive), the usability of the interface, and how well these features match the users' attention, motivation, interest, and need for aesthetic and sensory appeal, novelty, control, and challenge. Users must be made to feel a part of the interaction through an awareness of what the system is doing (via feedback), by feeling connected to the technology (via interactivity) or to other people, and by feeling in control over what is happening. *Disengagement* can occur for many reasons, such as the usability of the technology (ie, challenge and interactivity) and distractions in the user’s environment. This stage, depending on the outcome, will result in either positive (user’s needs and motivations are satisfied and they feel successful) or negative emotions (user feels frustration, overwhelmed by challenges or information, loss of interest or motivation). Users may cycle through the stages of engagement several times during a single session or over several sessions, thus demonstratating reingagement. Reingagement shares the same attributes as the point of engagement [30].

The aim of the current study was to explore participant engagement in the ManUp intervention using this framework and to generate directions for future research in the field. The behavioral outcomes of the ManUp randomized trial, relating to changes in diet and physical activity behavior, are forthcoming and will be published in a separate manuscript.

## Methods

### The ManUp Trial

**Figure 1:**
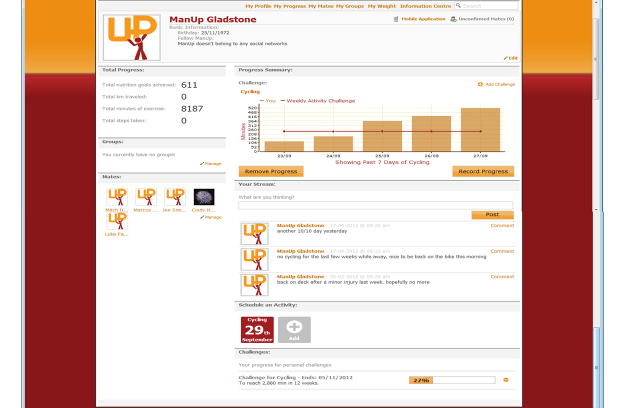
Screenshot of ManUP intervention website (participant profile).

A detailed description of the ManUp randomized controlled trials (RCT) protocol and study sample has been published previously [20]. Briefly, 317 middle-aged men (35-54 years old) recruited from two areas in Central Queensland, Australia were randomly allocated to one of two intervention arms: (1) an Information Technology (IT)-based intervention arm (Web- and mobile phone-based), or (2) a print-based positive-intervention control arm. A print-based intervention was selected as the positive control due to evidence that print-based interventions can be effective among adults, and because they are low-cost and wide-reaching and therefore may be of equal public health potential to IT-based approaches [31,32]. A positive intervention control was employed rather than a standard or true control (eg, wait list), as this approach has been recommended in order to more readily inform public health policy and practice [32,33]. Allocation was conducted on a 2:1 ratio in favor of the IT-based intervention. This was to control for attrition and ensure adequate power [20]. An overview of the interventions is provided below and a screenshot of the ManUp intervention can be found in Figure 1. This study was registered retrospectively (ACTRN12611000081910).

Both interventions comprised three main components: (1) educational materials that were designed to enhance health literacy by clearly communicating the health benefits associated with physical activity and a healthy diet, show the risks associated with inactivity and an unhealthy diet, and show the amount or type of physical activity and nutrition behaviors required to achieve health benefit; (2) ManUp physical activity, nutrition personal, and group “challenges” constructed to provide participants with specific, measureable, and time-based goals and to encourage self-monitoring behaviors, and; (3) self-monitoring resources, providing participants with the ability to record progress and keep themselves informed of progress towards completing these challenges.

Participants in the print-delivered intervention arm received these components via a hard copy booklet that contained a series of log sheets that could be used to monitor their progress and/or successful completion of any of the ManUp physical activity or nutrition challenges. Participants in the IT-delivered intervention arm were given access to a password protected website containing these components across six separate sections which participants could navigate (ie, My Profile, My Progress, My Mates, My Groups, My Weight, and Information Center). The website contained additional components that reflected the ability of the IT-based intervention to deliver automated feedback on challenge progress. Participants were also able to record and receive feedback on their body weight (kg), Body Mass Index (BMI) (kg/m2), and waist circumference (cm), as well as view summaries of all data recorded, schedule activities, search for “mates,” write on “mates” profile pages, and take part in group challenges. Additionally, participants with Internet access on their mobile phones were given access to a mobile phone application, facilitating self-monitoring behaviors by allowing them to quickly and conveniently start a new ManUp physical activity or healthy eating challenge, record progress, and view progress towards completing challenges.

### Recruitment

Before approaching ManUp participants an estimate of the required sample size to reach saturation was conducted [34]. As the study was relatively narrow in scope and based on participants’ direct experiences, we expected that the sample size needed to reach saturation would be small and aimed to recruit 30 ManUp participants (15 from each intervention arm) [34-36]. ManUp participants were contacted by telephone (in alphabetical order) and invited to take part in the study. Up to three call attempts were made to participants before labeling them as a nonresponder. It was intended that calls would continue to be made until 30 interviews had been conducted. However, after contacting 60 ManUp participants, with 20 participants consenting (a response rate of 33%), a decision was made to delay inviting additional men to participate until a judgment about theoretical saturation could be made on the already recruited sample. Theoretical saturation arises when no new data occurs after continuing sampling and analyzing data [36]. After conducting interviews with this sample, it was agreed that further interviews were unlikely to result in new information, and as such, no further attempts to recruit participants were made.

### Procedure

Semistructured telephone interviews were conducted and audiotaped by research assistants working on the ManUp project. During each interview, written notes were taken in as much detail as possible to aid in the interpretation of the recordings. Each interview ran for approximately 10 minutes. Telephone interviews were chosen over other interview methods due to the geographical dispersion of participants and evidence that this method can provide rich data [37].

Prior to conducting interviews with participants, training interviews were conducted on a convenience sample (n=5) to provide the interviewers with an opportunity to practice interview skills and refine the interview materials if necessary. Feedback was provided to the interviewers by the lead investigator (MJD) and necessary changes were made to the interview protocol prior to data collection.

### Discussion Guide

Interview questions were designed to explore attributes associated with participant engagement and disengagement in the intervention materials. The development of the open-ended questions was guided by O’Brien and Toms’ [30] conceptual model of engagement and centered on why participants took part in the study, what they liked and did not like about the intervention they received, and how they think the intervention could be improved. Leading questions about specific intervention attributes were avoided, and, instead, questions were designed to allow participants to voice their own views, values, and experiences. Participants were prompted to expand on their answers and give as many details as possible using standard prompting techniques (eg, requesting more information, paraphrasing, and using affirmative noises). The interview questions in the context of the conceptual model are presented in Table 1.

**Table 1:** Hypothesized attributes influencing engagement and related interview questions.

Phase	Hypothesised attributes influencing engagement	Interview questions
Point of engagement	Aesthetic appeal	What was the reason you participated in the study?
	Novel presentation	What did you expect to get out of the program?
	Users’ motivations	What did you like about the program?
	Users’ desired outcome/goal for interacting with the application	
	Users’ ability	
	Users’ perception that there is sufficient time to use the application	
Engagement	Usability of the interface	Did the program meet your expectations?
	Feeling of connectedness to the technology (influenced by physical, social and cognitive interactivity)	What did you like about the program?
	Feeling of control	
	Positive/negative emotions associated with how well the application features match the users’ motivation/goal, and need for sensory appeal, novelty and challenge	
Disengagement	Usability	What didn’t you like about the program?
	Distractions in the user’s environment	What would you suggest to improve the materials?
	Positive emotions (user’s needs are satisfied)	
	Negative emotions (frustration, overwhelmed by challenges or information, boredom, loss of motivation or interest).	

### Data Analysis

Data collection was conducted from April 2012 to June 2012. Data extraction was conducted between January and February 2013. Interview recordings were transcribed verbatim by a research assistant and analyzed thematically using a theory-driven code [38] into a tabular format (based on O’Brien and Toms’ [30] preexisiting description of attributes influencing engagement; see Table 1). During the coding process frequencies of each issue raised were noted. Data were analysed in this way to enable the investigators to identify patterns and facilitate discovery of the most prevalent themes. However, in accordance with qualitative research methodology, where the focus is on richness and not representativeness, frequencies are not reported in the text [35,36]. There were two researchers (CS, MWD) that conducted the coding process independently to ensure the trustworthiness of the themes. Results of the independent analyses were compared and discussed until agreement was reached.

## Results

### Participants

Twenty out of the 60 individuals contacted took part in the study. A summary of participant characteristics is presented in Table 2 (data obtained using Web-based surveys as part of baseline assessment in the ManUp study) [20]. Overall, the majority of participants were professional or white collar workers, with moderate health literacy for physical activity and nutrition topics, were classified as overweight or obese (based on BMI), and were partaking in some physical activity, but not at a level sufficient to meet the national guidelines (ie, 150 minutes/week of moderate-vigorous physical activity spread across at least five days) [39]. There were no significant differences between ManUp participants who participated in the RCT and those who participated in the current study in terms of age, income, health literacy, physical activity, and diet behavior (*P*>.05). However, a higher proportion of interviewed participants were employed in professional and white collar occupations than those who were not interviewed (*P*=.03), and of those that received the IT-based intervention, interview participants of the current study had higher median log-in rates as compared to those who did not participate (*P*=.01).

Themes from the theory-driven analysis are summarized here. Representative quotes relating to each theme are provided in Tables 3 and 4.

**Table 2:** Participant characteristics.

		Print (N=7)	IT-based (N=13)
**Demographics**			
	Age (years; x, SD)	43.42 (6.02)	42.08 (4.25)
	University education (N)	6	4
**Employment type (N)**			
	Professional	4	9
	White collar	2	4
	Blue collar	1	0
BMI		27.69 (2.81)	31.33 (7.56)
**Knowledge**			
	Nutrition literacy scale (x, SD)	25.85 (1.46)	25.58 (1.67)
	Physical activity literacy scale (x, SD)	2.57 (1.81)	3.66 (1.30)
**Behavior**			
	Meeting the physical activity guideline (N)	3	7
	Moderate/vigorous physical activity (mins/wk; x, SD)	42.86 (57.07)	123.33 (278.25)
	Serves of vegetables/day (x, SD)	3 (1.91)	2 (1.34)
	Serves of fruit/day (x, SD)	2.71 (2.62)	2.41 (2.27)
	Frequency of eating red meat last week (x, SD)	6 (0.81)	5.41 (1.62)
**Usage data**			
	Median log-ins (1-36 weeks) and interquartile range	N/A	13.5 (interquartile range=26)

### Users’ Motivations

The most common motivating factors among participants were weight loss and gaining the necessary tools and incentives to self-manage one’s health. In particular, participants expressed a desire to “gauge” their health and fitness levels and to be provided with feedback and advice on how to control their weight and maintain healthy lifestyle habits. For a few participants, participation was driven by external factors, such as pressure to participate from their partner.

### Users’ Desired Outcomes

Overall, the outcomes people anticipated from engaging in the program largely reflected their motives for participating. Most were expecting to receive guidance and counseling from the project team to help them enhance their diet and participation in physical activity. For some, this guidance and advice were expected to be specific, such as a prescriptive diet plan to follow or support for their particular sport and activities. For others, the type of advice and support expected were described more generally, such as “tips and suggestions” to live a healthier life. Strategies to help participants stay disciplined and to take action were also expected, such as the provision of materials to record diet and physical activity behaviors. A few participants also expressed that they expected to improve their lifestyle behaviors and/or weight status as a result of participating in the study.

### Users’ Positive Emotions

Participants from both intervention groups liked that the information they were provided with was easy to read, use, and had an appropriate tone (ie, not derogatory). Participants from both groups liked that the materials could be used as a benchmark and reference tool when thinking about their own health. Participants who received the IT-based intervention liked the ability to record and view a visual summary of their progress.

### Users’ Negative Emotions

Some participants who received the printed information found that the booklet was too long and that some of the text was long-winded. Furthermore, there were a few participants who expressed disappointment with the level of interaction and feedback provided and felt they would have done better with the “Web-based stuff.” Among those who received the IT-based intervention, some participants expressed that they would have liked functional aspects of the website to be improved, such as the ability to enter and keep track of different types of activities, the ability to enlarge text, and the progress calendar. Furthermore, a few participants raised sustaining self-monitoring as an issue, especially when personal physical activity routines did not change (ie, self-monitoring via the website was considered less useful) or when in out-of-service areas (ie, when self-monitoring could not be done immediately and conveniently due to a lack of Internet connection). A few participants also expressed disappointment with the intervention content, with some participants reporting that the physical activity and nutrition content was not prescriptive enough, and others reporting that they would have liked to have received more personalized information and feedback about how their changes in health behavior were likely to impact on their health.

### Attributes Desired by Users

Suggestions on how to improve the print-based intervention included providing more tips and helpful hints that are based on the experiences of their peers, and transferring the intervention onto a Web or mobile phone platform to make it more interesting and accessible. Suggestions on how to improve the IT-based intervention were more varied and included both suggestions on how to improve the website usability and for improving intervention content. Specific functional components requested by participants included a facility to report IT-based issues, reminder emails offering direct links to participant profiles (without logging in), the capacity to use the mobile app when there is no Internet connection, and to sync the data with the website at a later date. Suggestions on how to improve intervention content included providing links to nutrition and physical activity information on a separate page of the website, allowing participants to set their own challenge metrics, providing more detailed and iterative feedback, and providing access to other useful tools, such as a calorie conversion calculator. Some of these suggestions, namely a facility to report IT-based issues and links providing further lifestyle information, were actually included on the website.

**Table 3:** Representative quotes from participants relating to each theme.

Theme	All participants
Users’ motivations	I was looking to lose a bit of weight
	I was hoping you could give me some sort of insight into how to control my weight
	My wife told me I had to
	Just to gauge my fitness
	To keep track that I’m doing the right thing and a bit more of an incentive
Users’ desired outcomes	To gain a bit more knowledge on my body and how I can better manage my health
	Guidance to make sure that I was doing the right thing as far as exercising a bit more and eating properly
	To record what I was doing and then talk to your consultant and actually see ways of improving either fitness or health
	A solution to weight loss
	Documenting what I was doing/motivation to continue

**Table 4:** Representative quotes from participants relating to each theme.

	Print	IT-based (Web + mobile)
Users’ positive emotions	I didn’t know too much about what I was doing each day so that helped me out	The program made me focus more on my physical activity and diet after actually seeing the data
	I went in with an open mind and it was pretty much what I thought it would be	Overall I think it’s great
	It was easy to read	I got a little bit out of it. It actually encouraged me to start walking a lot more. In my particular job I'm out of town a lot so there are not a lot of regular exercise programs I can actually sign up for. Whereas, I ended up buying myself a pedometer and I have been walking
	Gives you a good benchmark on how to lose weight	It was easy to read
	I used print material as a reference tool	I really liked seeing the visual record of my progress
	They were well written and It wasn’t too derogatory or you didn’t feel like you were being taught a kindergarten lesson but by the same token, it was quite readable and achievable for anybody with limited literacy skills	I like the idea of being able to use the calendar to record progress
	It was all pretty good relevant information	I’m not high tech minded but I could still use it
Users’ negative emotions	I was expecting more feedback and interaction	I wanted to be told what the outcome would be if I did specific amounts of activity
	I think I would have done better with the Web-based stuff, more motivation that way	I wanted to count cross training exercise but didn’t think that it was really designed for this
	I was never going to be able to fill the whole book in and I suppose you’ve written the book with that in mind, so I suppose the expectation might have been a little bit high for me to fill something in every week	I didn’t like that you couldn’t enlarge the calendar
	They were a little bit long winded	It wasn’t prescriptive enough
		It didn’t give me the outcome I was looking for
		I think I may have let the system down by not following through as much as I should. Initially I was recording my activity weekly but it was a routine that didn’t change much so I sort of fell off on the updating sort of sense
Attributes desired by users	Tips, helpful hints	I would have liked reminder emails with link to direct entry without log-in
	Encouragement, people to give you ideas	Capacity to enter data when no reception is available and then sync when phone has reception
	I think one method to address some of my habitual failings, is if it was on either a mobile phone app or an Internet version. Cause I’m more of a technology orientated person than I am paper orientated. I probably would have addressed and achieved more of the challenges purely because, you know, if it’s on the phone or on my computer it’s more ah, it’s more interesting and more accessible for me	Post challenge-report that detailed progress over time period of the entire challenge
		I would have liked to have been able to measure total physical activity across different types of activity
		I’d have liked a calorie conversion option on my progress chart
		I would have liked to have been able to set my own challenge metrics and timeframes
		I’d like to be able to pause my challenge or reset for when I am sick or away

## Discussion

### Aim of the Study

Poor user engagement in Web-based health behavior interventions is consistently reported in the literature [14,28]. Despite this, very little research in the field has explored user experiences in human-computer interactions and how such experiences may relate to engagement, especially among males who are in general harder to engage than women in behavior change interventions [9,10]. The aim of the current study was to explore participant engagement in the ManUp intervention using a conceptual framework [30] and to inform future research in the field. Although not conclusive, the current study provides some insight into what personal attributes of middle-aged males (such as their key motives and goals for participating) and attributes of the intervention materials (such as usability, control, and interactivity) may impact on user engagement in this group. When considered in the context of previous research, these findings will be helpful for informing the design and implementation of future health behavior interventions for males and assist researchers and practitioners to engage males in health behaviors.

### Comparison to Previous Research

The desirable intervention components reported in this study are similar to those reported in previous (atheoretical) qualitative studies exploring participant’s experiences with Web-based interventions [40,41]. Participants in Morgan et al’s [40] Australian self-help, exercise, diet, and information technology (SHED-IT) intervention, an Internet-based weight loss program designed specifically for men [41], found the use of a calorie converter website that provided instant and visual feedback an invaluable part of the program, especially for education about foods and for self-monitoring. Participants also reported that email feedback was helpful, but felt they would have benefited more if the information provided was more specific to them personally. SHED-IT participants were not interested in the forum facility of the website, which was designed to provide them with an opportunity to engage in discussions with other men about weight loss. Participants in Ferney and Marshall’s [42] study (including both males and females) reported that the overall usability of the website was extremely important to them and that engagement in the website would be better sustained if there were more interactive features such as self-reported progress charts allowing users to set goals and monitor their progress, and if they received regular newsletters via email. They also suggested that a forum would help encourage engagement by offering a social support network along with other helpful advice and suggestions. Interestingly, in randomized trials associated with both of these previous qualitative studies, participants did not use forum features of the website [41,43]. The current study did not include a forum feature, rather the ability to post comments on their own or another user's profile page was included, and this was not widely used by participants. It may be that utilization of the forum is dependent on social environmental factors such as the presence of a forum moderator for participants to interact with. In the current study, the low usage of the comments feature may be due to participants not knowing each other and being reluctant to comment or interact with unknown people. Future research is needed to explore this further. One website component that was not discussed by participants in the current study, but did emerge as an important factor in both of the aforementioned studies, was the speed of the website. In both of these previous studies, participants reported dissatisfaction and reduced use of the website if the loading time was slow [27,28].

### Implications for Practice

The findings of this study provide some insight into how we may improve engagement among males in Web-based behavior change interventions targeting physical activity and nutrition. First, as weight management appears to be a key motivator for many males participating in Web-based programs, participant engagement (in terms of recruitment and content) may be higher if weight loss is also highlighted as an intervention outcome. Findings from the SHED-IT trial showed that how the weight loss intervention is framed is important and suggest that programs that are framed as nonintrusive and flexible are most appealing [27]. Second, as participant’s experience of the intervention (positive and negative) seemed to be guided by their initial expectations, managing outcome expectations from the outset and avoiding violations of these expectations may help to improve engagement, retention, and maintenance [30,31]. This could be achieved in the following ways: (1) by ensuring recruitment information adequately explains the aim, content, and structure of the intervention program; (2) by educating participants about the health outcomes associated with adhering to the intervention protocol; (3) by allowing participants to set their own goals; and (4) by ensuring participants set goals that are both achievable and in line with their desired outcomes. The use of positive reinforcement (internal or external) may also be helpful, especially in cases where outcomes may not be immediate (eg, disease prevention) [30,31]. Finally, participants provided several suggestions regarding how to improve website usability and intervention content which may improve efficacy, engagement, and maintenance in future interventions. As some of these suggestions (namely a facility to report IT-based issues and links providing further lifestyle information) were actually included on the website, this may suggest potential user navigation issues, communication issues (eg, use of the word “bug” for computer issue), lack of use of the IT-based platform, and/or that the website did not sufficiently “grab” the attention of some participants to encourage more elaborate use of the site. These issues highlight that researchers and practitioners need to consider the attributes of user disengagement (as well as engagement) when designing websites. This is particularly so for disengagement that occurs due to negative emotions, such as feeling frustrated due to usability issues with the website or feeling bored due to lack of interactivity and/or novelty of the content.

### Strengths and Limitations

A major strength of this study is that it provides a greater understanding of what males want in terms of Web-based health behavior interventions. Males are a hard-to-reach population [9,10]. As such, little male specific research has been undertaken and best practice intervention approaches targeting males have not been identified. This research directs some insight into what recruitment and intervention strategies may enhance a male’s engagement, retention, and maintenance in these interventions. This is useful information, considering the paucity of research in this area, a male’s preference for IT-based strategies compared to face-to-face strategies, and the potential public health impact of such interventions if engagement and maintenance can be achieved. However, there are some limitations that should be acknowledged when interpreting these findings. As not all participants agreed to be contacted to take part in follow-up interviews, the results presented in this manuscript may not be representative of the whole ManUp sample. In addition, the ManUp Trial participants were primarily employed in white-collar and professional occupations, as were the participants of the current study. As such, the findings may not be representative of those in lower socioeconomic positions. While interesting outcomes have been reported, due to the exploratory and qualitative nature of the study (where the focus is on information richness rather than representativeness) [21,22], more comprehensive methodologies, such as intervention research, are now needed to confirm the outcomes revealed by the present study.

Compared to their female counterparts, males have higher age-specific rates of chronic disease (such as heart disease, Type 2 diabetes, obesity, and mental health issues) [11-13] and are less likely to engage in lifestyle modification programs and utilize health services [9,10]. Given this, interventions that appeal to males, which meet their specific needs and that are not resource intensive are required. The development and evaluation of lifestyle modification programs that have a strong theoretical and evidence base, grounded in sound behavioural and communication-based research, and that are tailored to specific at risk subgroups are an important public health response to the growing prevalence of chronic disease and the survival deficit experienced by males. Our study highlights intervention and participant attributes that impacted on the engagement of males participating in a lifestyle modification program. These findings will help inform the design and implementation of future health behavior interventions for males and assist researchers and practitioners to engage males in health behaviors.

## Abbreviations

BMI: body mass index
IQR: interquartile range
IT: information technology
RCT: randomized controlled trials
SHED-IT: self-help, exercise, diet, and information technology
